# Clinical practice guidelines for neonatal hypoxic-ischemic encephalopathy: A systematic review using the appraisal of guidelines for research and evaluation (AGREE) II instrument

**DOI:** 10.3389/fped.2023.1092578

**Published:** 2023-03-22

**Authors:** Yasser S. Amer, Jasim Anabrees, Mohamed Abdelmawla, Ayman Abdalgader, Asmaa Almazroei, Ibrahim Alhifzi, Abdullah Hawash AlOnazi, Yasser Sabr, Layal Hneiny, Ahmed El-Malky, Ayesha Alshalawi, Ahmed Alayoubi, Iftikhar A. Chaudhry, Omar Elkhateeb

**Affiliations:** ^1^ Pediatrics Department, King Khalid University Hospital, King Saud University Medical City, Riyadh, Saudi Arabia; ^2^ Clinical Practice Guidelines and Quality Research Unit, Quality Management Department, King Saud University Medical City, Riyadh, Saudi Arabia; ^3^ Research Chair for Evidence-Based Health Care and Knowledge Translation, King Saud University, Riyadh, Saudi Arabia; ^4^ Alexandria Center for Evidence-Based Clinical Practice Guidelines, Alexandria University, Alexandria, Egypt; ^5^ Adaptation Working Group, Guidelines International Network, Perth, Scotland; ^6^ Pediatrics Department, College of Medicine, King Saud University, Riyadh, Saudi Arabia; ^7^ Saudi Neonatology Society (SNS), Riyadh, Saudi Arabia; ^8^ Pediatrics Department, King Fahad Armed Forces Hospital, Jeddah, Saudi Arabia; ^9^ Neonatology Department, Pediatric Hospital, King Saud Medical City, Riyadh, Saudi Arabia; ^10^ The Specialist Hospital-Abha, Abha, Saudi Arabia; ^11^ Neonatal Intensive Care Unit, Critical Care Services, King Fahad Medical City, MOH, Riyadh, Saudi Arabia; ^12^ Obstetrics and Gynecology Department, College of Medicine, King Saud University, Riyadh, Saudi Arabia; ^13^ Wegner Health Sciences Library, University of South Dakota, Sioux Falls, SD, United States; ^14^ Morbidity and Mortality Unit, King Saud University Medical City, King Saud University, Riyadh, Saudi Arabia; ^15^ Public Health and Community Medicine Department, Theodor Bilharz Research Institute (TBRI), Academy of Scientific Research, Cairo, Egypt; ^16^ Nursing Department, King Saud Medical City, Ministry of Health, Riyadh, Saudi Arabia; ^17^ Pediatric Department, Umm Al Qura University, Makkah, Saudi Arabia; ^18^ Obstetrics and Gynecology Department, Al-Yamamah Hospital, Ministry of Health, Riyadh, Saudi Arabia

**Keywords:** hypoxic ischemic encephalopathy, HIE, pediatrics, neonatology, clinical practice guidelines, systematic review, AGREE II instrument, quality assessment

## Abstract

**Background and Objective:**

To systematically review, critically appraise the quality of recent clinical practice guidelines (CPGs) for neonatal hypoxic ischemic encephalopathy (HIE), and map their recommendations.

**Data Sources:**

CPG databases (GIN, ECRI, NICE, SIGN, DynaMed), Bibliographic databases (PubMed, Embase, CINAHL), and related specialized professional societies (e.g., AAP, CPS, BAPM, RCPCH, and SNS).

**Study Selection:**

Original de-novo developed evidence-based CPGs for HIE, group authorship, Arabic or English languages, and international or national scope. The systematic review was drafted according to the Preferred Reporting Items for Systematic reviews and Meta-Analyses (PRISMA) statement and Johnston et al methodological guide.

**Data Extraction:**

Quality assessment of the included HIE CPGs by the Appraisal of Guidelines for REsearch & Evaluation II (AGREE II) Instrument and report their characteristics, AGREE II ratings, and recommendations

**Data Synthesis:**

Our search retrieved 2,489 citations, of which two recent HIE CPGs were eligible and appraised: Canadian Paediatric Society (CPS) and Queensland Maternity and Neonatal Services (QMN). The overall assessment of the QMN CPG was superior (83%). Domain 1 (Scope & Purpose) scored (47%, 63%), Domain 2 (Stakeholder Involvement) (72%, 39%), Domain 3 (Rigour of Development) (48%, 43%), Domain 4 (Clarity & Presentation) (100%, 96%), Domain 5 (Applicability) (59%, 9%), and Domain 6 (Editorial Independence) (67%, 17%) for the QMN and CPS CPGs respectively. All appraisers recommended the QMN CPG for use in practice.

**Conclusion:**

The methodological quality of the QMN CPG was superior with the relevant recommendations for its use in neonatal practice.

**Limitations:**

limited to Arabic and English languages.

**Systematic Review Registration:**

https://www.crd.york.ac.uk/prospero/display_record.php?RecordID=258291, identifier: CRD42021258291.

## Introduction

Survivors of hypoxic-ischemic encephalopathy (HIE) have shown reduced neuropsychological functioning, behavioral adjustment, and school outcomes during adolescence among other neonatal morbidities, mortalities, and outcomes (1).

Several initiatives were launched to promote and advance evidence-based neonatal healthcare by national (e.g., American Academy of Pediatrics, British Association of Perinatal Medicine, and Saudi Neonatology Society) and international organizations and professional societies [e.g., International Society for Evidence-Based Neonatology (EBNEO)] (2).

Despite the emphasized potential of Clinical Practice Guidelines (CPGs) to optimize patient outcomes and clinical practice, an increasing volume of CPGs is being published of variable quality in the field of neonatology (3–5).

In 2012, The Saudi Neonatology Society (SNS) published a CPG for Whole Body Cooling for infants with HIE (6). The SNS has recently launched a number of national projects to adapt evidence-based CPGs for the management of high-priority health topics in neonatal healthcare using the “KSU-Modified-ADAPTE” as a formal CPG adaptation methodology, with the goal of providing evidence-based guidance and recommendations to neonatologists and pediatricians across the country (7–11). As newer evidence and CPGs were published overtime, SNS decided to update this CPG and launch a national HIE CPG project.

The systematic review (SR) and quality appraisal of CPGs using the Appraisal of Guidelines for REsearch & Evaluation II (AGREE II) Instrument is a critical step in the KSU-Modified-ADAPTE CPG adaptation process (12, 13).

The overarching CPG adaptation project was registered in the *PREPARE (Practice guideline REgistration for trancPAREncy)* platform that is hosted by the University of Lanzhou in China http://www.guidelines-registry.org/ (Registration Number: IPGRP-2021CN384) (14).

This study aimed to report the systematic review and quality assessment of HIE CPGs as a part of the HIE CPG adaptation process.

## Methods

The protocol for this systematic review of CPGs was registered in PROSPERO (International Prospective Register of Systematic Reviews) (ID: CRD42021258291) (15). This systematic review was guided by the Preferred Reporting Items for Systematic reviews and Meta-Analyses (PRISMA) statement in addition to the methodological guide proposed by Johnston et al. (12, 15).

Our Guidelines Review Group (GRG) included seven expert consultant neonatologists: one of them with expertise in systematic reviews, two consultant Obstetricians and Gynecologists, a senior nurse, a medical and healthcare librarian, and a CPG methodologist with a background in pediatrics.

### Data sources and search strategy

The medical librarian systematically searched MEDLINE, EMBASE, and CINAHL databases for relevant CPGs using the Ovid platform and hand-searched the relevant CPG databases and repositories for eligible CPGs (Supplementary information).

Two reviewers (MA and OE) conducted the title and abstract screening of the CPGs and articles independently. Two different reviewers double-checked the full-text screening (YSA and JA) and disagreements were resolved through focus group discussions. The full inclusion and exclusion criteria were reported in the PROSPERO protocol (16). The search and screening for eligible HIE CPGs were updated before the submission of the manuscript.

### AGREE II appraisal of the eligible HIE CPGs

Three members of the GRG attended capacity building training in AGREE II appraisal of CPGs. The AGREE II Instrument (www.agreetrust.org) has 23 items or questions divided into six domains: scope and purpose, stakeholder involvement, rigor of development, clarity of presentation, applicability, and editorial independence. Using a 7-point Likert scale, each item was scored using its online platform (My AGREE PLUS). Each CPG was critically appraised by four AGREE II raters including four clinicians, and one of them was a CPG methodologist (13).

### Data analysis plan

For each AGREE II domain, we determined standardized scores ranging from 0% to 100% using the techniques advised by the AGREE II instrument's equations. A comparison tabular style was used to summarize the main recommendations of the applicable CPGs (17).

### Inter-rater analysis

For each item in each area of the two assessed CPGs, we performed inter-rater reliability tests to gauge the degree of agreement between raters (IRR). We did this by utilizing a percent agreement inter-rater reliability assessment test. The consistency of ratings or the capacity for datasets that were gathered as clusters or sorted into clusters using intra-class correlation were also assessed in the second overall assessment (OA2) in addition to the percent agreement in the first overall evaluation (OA1). One common IRR strategy is intra-class correlation (ICC).

When there are more than two raters, we use this. Standards from the same set appeared to be fairly comparable, as shown by a strong intra-class correlation coefficient (kappa) around 1. A low kappa score near 0 denoted a lack of similarity between standards from the same collection. Since our raters and rates varied, we used ANOVA “One-Way Random” on SPSS Statistics, version 21. The extensive range of numerical data from groups or clusters is why we chose ICCC. This allowed us to assess the repeatability and the degree to which peers shared particular characteristics. We looked at how well two categories on an ordinal scale agreed with one another.

Given that the data originated from an ordered scale, we employed weighted kappa (quadratic weights). Following are the weights' calculations: The notation is Cohen's kappa. We decided on linear weights because the difference between the first and second categories was similar to the difference between the second and third categories, and so on. The kappa (K) statistic is used to measure agreement (18, 19): When categorization systems agree completely, K = 1 when there is no agreement greater than chance, and K is negative when there is agreement worse than chance. (Supplementary information) demonstrates possible interpretations of the K value (20).

## Results

**Figure 1:**
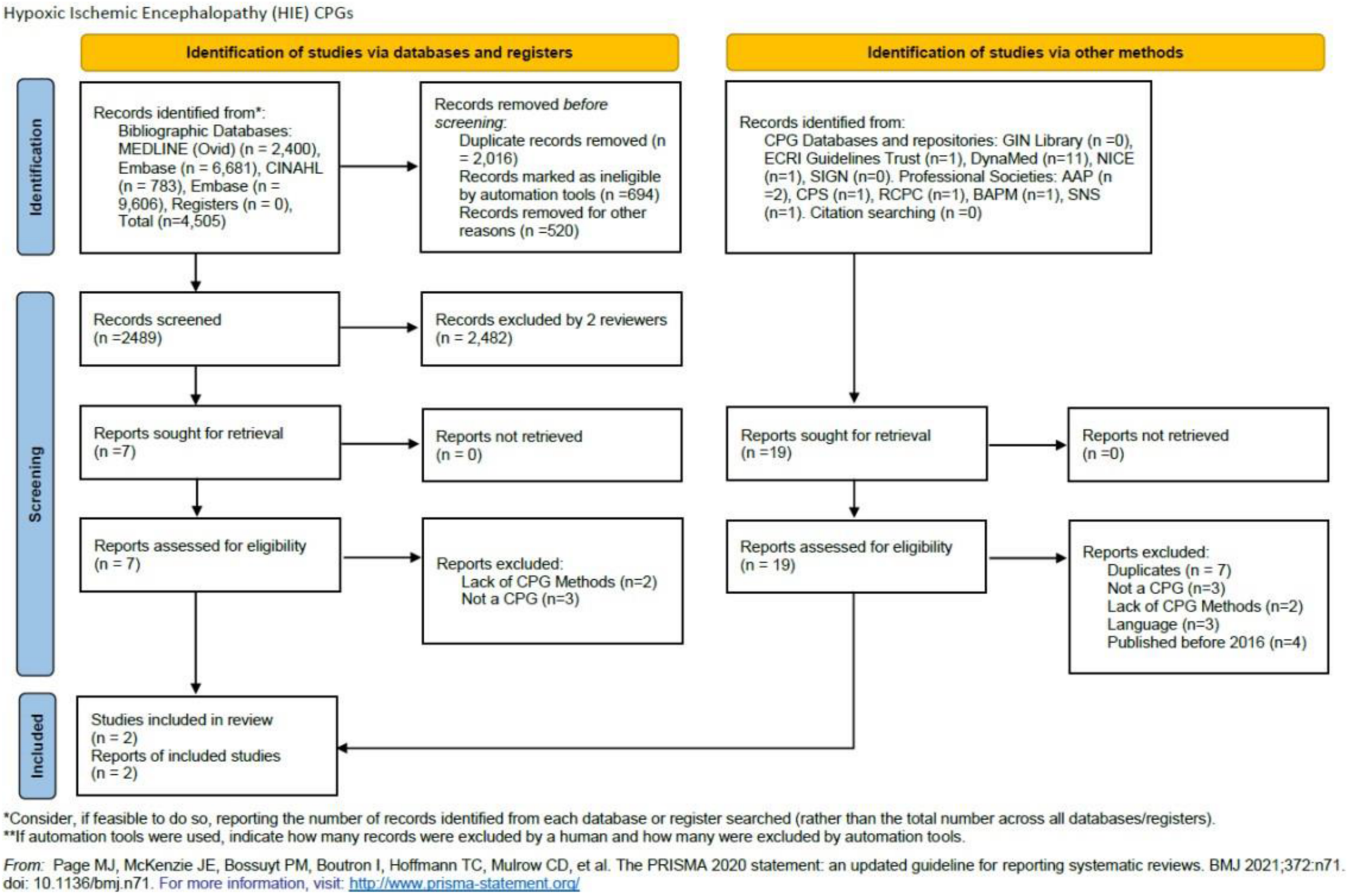
PRISMA 2020 flow diagram for new systematic reviews which included searches of databases, registers, and other sources.

A total of 4,505 records were retrieved from bibliographic databases and 19 from CPG databases and professional societies. After the title, abstract, and full-text screening using Rayyan https://www.rayyan.ai/ only two source original CPGs were found to be eligible for the AGREE II quality assessment step. Two reviewers conducted the screening (OA, MA) and two additional reviewers (JA, YSA) resolved any discrepancies through discussions (Figure 1).

### Key characteristics of HIE CPGs

Table 1 highlights the characteristics of all eligible CPGs. The CPG developer organizations were reference, professional organizations in pediatrics, neonatology, or general non-specialized including CPS, and QH. Both organizations were from high-income countries.

**Table 1:** Characteristics of included HIE CPGs.

Organization, Category, Country (Health System, Economic classification)	Scope	CPG Title	Year of publication	No. of references (No. of systematic reviews cited)
Queensland Clinical Guidelines Steering Committee and Statewide Maternity and Neonatal Clinical Network (Queensland), Australia (QMN), Governmental Organization *(National Health Insurance, High- Income)* (21)	Statewide	Hypoxic-ischaemic encephalopathy (HIE), Maternity and Neonatal Clinical Guideline, Queensland Clinical Guidelines	2021	96 (10)
Canadian Paediatric Society, Canada (CPS), Professional Society *(National Health Insurance, High- Income)* (22)	National	Hypothermia for newborns with hypoxic-ischemic encephalopathy	2018	72 (2)

## Reporting the quality of HIE CPGs

### Domain 1: scope and purpose (items 1–3)

The two CPGs (CPS, QMN) scored (63%, 47%) respectively in domain 1 where the CPS CPG reported its objectives, health questions, and patient population more clearly addressing most of the AGREE II criteria and additional considerations.

### Domain 2: stakeholder involvement (items 4–6)

CPS and QMN CPGs scored 39% and 72% respectively, where the QMN CPG development group was properly reported and included a multidisciplinary team representing all related specialties to neonatal HIE.

### Domain 3: rigor of development (items 7–14)

CPS and QMN scored 43% and 48% respectively. There was a large area for improvement in both CPGs in properly and clearly reporting the search methods, evidence selection criteria, strengths and limitations of the evidence, formulation of recommendations, consideration of benefits and harms, link between recommendations and evidence, external review, and updating procedure. Both CPGs reported an overall process of their CPG development the aforementioned criteria need to be reported transparently in clear detail to facilitate the replication of the CPG development process. Both CPGs have not reported using the GRADE (Grading of Recommendations; Assessment; Development and Evaluations) Method.

### Domain 4: clarity of presentation (items 15–17)

CPS and QMN CPGs scored 96% and 100% in domain 4 respectively. Both CPGs presented specific and unambiguous recommendations, comprehensive management options, and a set of identifiable key recommendations.

### Domain 5: applicability (items 18–21)

CPS and QMN CPGs scored 9% and 59% in domain 4 respectively. The QMN CPG was superior in its variable set of CPG implementation tools inside the CPG article or on the website including management flowcharts and posters, points for discussion with the parents, checklist for therapeutic hypothermia, assessment of encephalopathy severity (Sarnat scoring), Sarnat and Sarnat staging of HIE, educational material, and patient information on HIE.

### Domain 6: editorial independence (items 22, 23)

CPS and QMN CPGs scored 17% and 67% respectively where QMN CPG documented the funding body and the conflicts of interest more clearly.

### The first overall assessment

The AGREE II standardized domain scores for the first overall assessment was higher for the QMN CPG (83%) than the CPS CPG (63%).

### The second overall assessment or recommending the CPGs for use in practice

The second overall assessment revealed a consensus between the four reviewers on recommending the use of the QMN CPG while half of the reviewers recommended using the CPS CPG and half of them recommended its use with modifications.

### Inter-rater analysis

**Figure 2:**
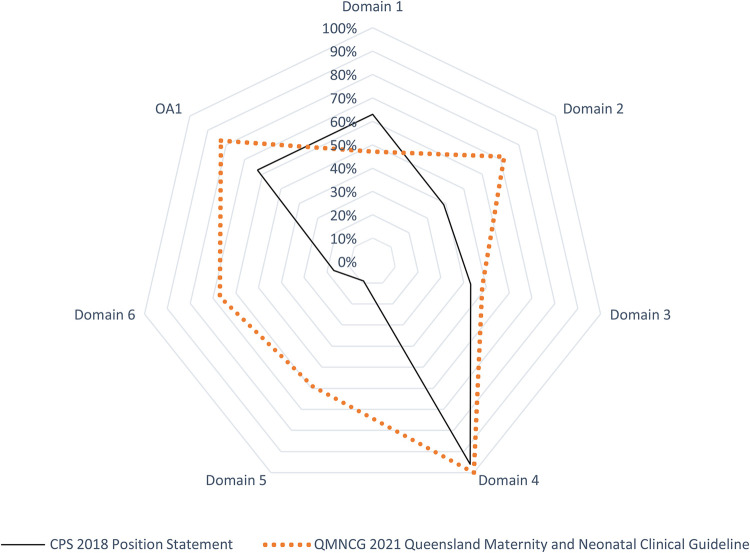
HIE CPGs/AGREE II domains: standardized Items’ scores.

**Figure 3:**
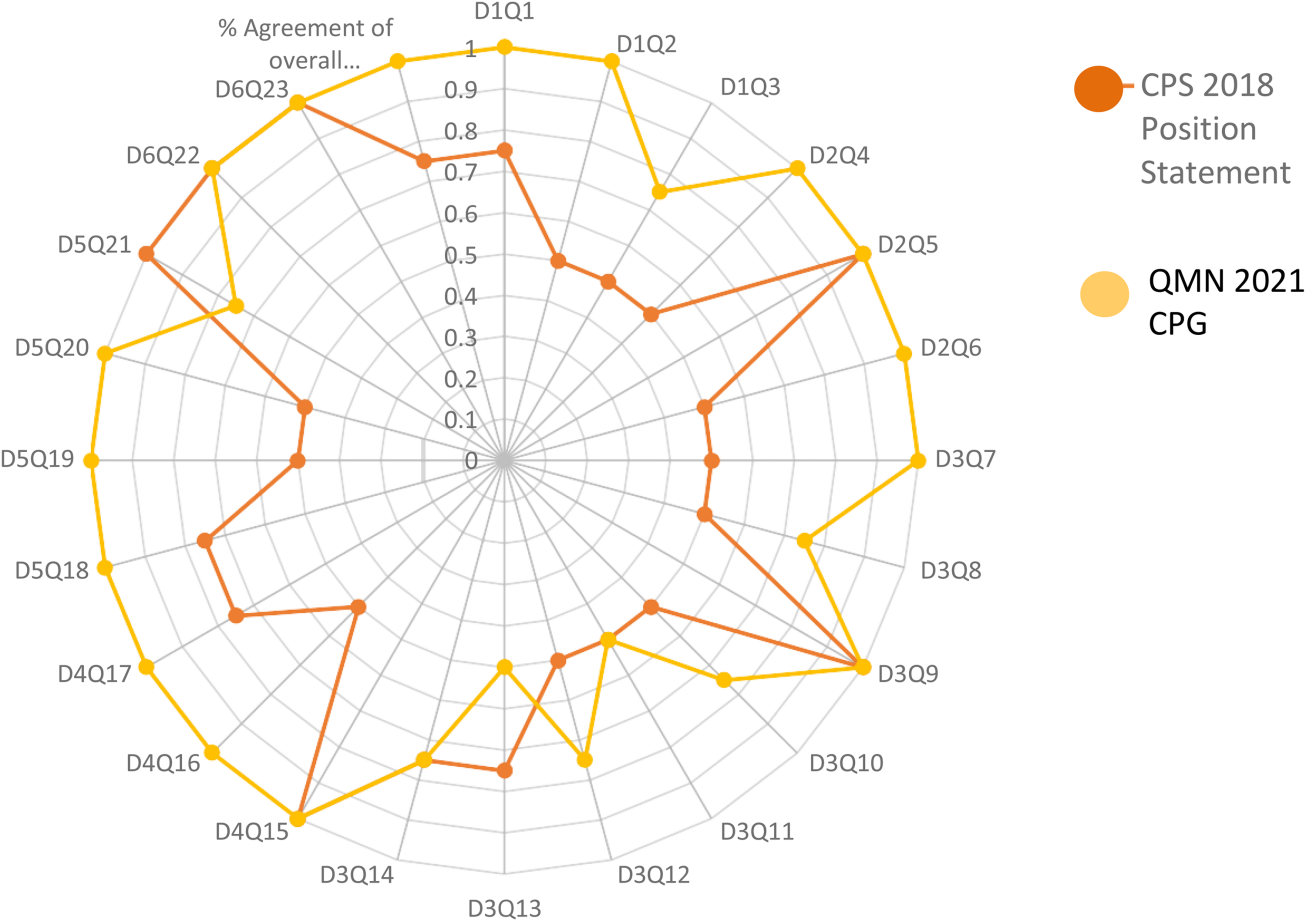
Showed percent agreement among raters for the two HIE practice guidelines focusing on every question in every domain.

In terms of Classification of the strength of agreement among the four raters against the two guidelines; the assessment was classified according to strength into Poor, Fair, Good, Very Good, and Excellent. With an emphasis on the sum of scores, the sum of OA scores, and the first overall assessment. Regarding the CPS 2018 Position Statement; it was arranged as follows; 0, 0, 12, 6, 6, 333, and 19 however, QMNCG 2021 Queensland Maternity and Neonatal Clinical Guideline was 0, 0, 2, 6, 16, 431 and 24. Supplementary Tables S2.1 and S2.2. shows the intraclass correlation coefficient (Kappa value) among raters for the two CPGs for the second Overall Assessment. The number of observed agreements is six (77.16% of the observations). 7.0 agreements are predicted by chance (85.00 percent of the observations). Kappa = 0.912; kappa SE = 0.586; 95% confidence interval: Weighted Kappa = 0.081 for values ranging from 0.752 to 1.642 (Figures 2, 3).

### Recommendation mapping of the appraised CPGs

Table 2 shows a map of the recommendations from both HIE CPGs (21, 22).

**Table 2:** Recommendation mapping for both appraised CPGs for HIE (21, 22).

**Management Point**	**CPS HIE CPG** **2018**	**QMN HIE CPG *(updated)*** **2021**
**Inclusion Criteria**	– Term and late preterm infants ≥36 weeks GA with HIE who are ≤6 hours old and who meet either treatment criteria A or treatment criteria B, and also meet criteria C: A. Cord pH ≤7.0 or base deficit ≥−16, OR B. pH 7.01 to 7.15 or base deficit −10 to −15.9 on cord gas or blood gas within 1 h AND 1. History of an acute perinatal event (such as but not limited to cord prolapse, placental abruption, or uterine rupture) AND 2. Apgar score ≤5 at 10 minutes or at least 10 minutes of positive-pressure ventilation C. Evidence of moderate-to-severe encephalopathy, demonstrated by the presence of seizures OR at least one sign in three or more of the six categories or criteria for defining moderate and severe encephalopathy.	– Greater than or equal to 35 weeks’ gestational age. – Birth weight greater than or equal to 1,800 g – Able to begin cooling before 6 hours of birth – Evidence of perinatal/intrapartum hypoxia, as indicated by at least one of: ○ Apgar score of less than or equal to 5 at 10 minutes ○ Needing mechanical ventilation or ongoing resuscitation at 10 minutes ○ pH less than 7.00 or a base excess worse than or equal to minus 12 mmol/L on cord/arterial/venous/capillary blood gas obtained within 60 minutes of birth – Evidence of moderate or severe encephalopathy
**Exclusion Criteria (or Contraindications)**	– Moribund infants or infants with major congenital or genetic abnormalities for whom no further aggressive treatment is planned; infants with severe intrauterine growth restriction; infants with clinically significant coagulopathy; and infants with evidence of severe head trauma or intracranial bleeding	– Major congenital abnormalities identified including: ○ Suspected neuromuscular disorders or Suspected chromosomal abnormalities ○ Life-threatening abnormalities of the cardiovascular or respiratory systems – Uncontrolled pulmonary hypertension – Critical bleeding or coagulopathy – Severe head trauma or intracranial bleeding – Baby is moribund or so severely affected that there is little hope for a normal outcome i.e. moribund or “in extremis” (e.g. very low BP or severe acidosis unresponsive to treatment)
**Continuous Electroencephalogram (EEG)/ or Amplitude-integrated Electroencephalogram (aEEG)**	– When available, assessment with an aEEG for at least 20 minutes to document abnormal tracings or seizures, particularly for infants with moderate encephalopathy, may be useful for determining eligibility. – However, at the time of this CPG publication (2018), there were insufficient data to make this recommendation.	– If available, commence continuous aEEG for 96 hours or EEG with simultaneous video recording (if available) to confirm clinical seizures, detect subclinical seizures, and assess background – Both conventional EEG and aEEG provide information about the severity of HIE and perform well in predicting outcomes.
**Fluid management**	– Not Mentioned.	– IV 10% glucose at 40–50 mL/kg/day
**Feeding**	– Early minimal enteral feeding (10 mL/kg/day to 20 mL/kg/day) during hypothermia, initiated during the first few days of life, is safe and feasible for newborns with HIE – more than minimal feeds is not as safe because gut perfusion may be decreased during cooling	– Do not feed if receiving therapeutic hypothermia – Cautiously reintroduce feeds following rewarming: breast milk is ideal
**Resuscitation**	– Not Mentioned.	– Babies typically require respiratory support (Continuous positive airway pressure (CPAP) or positive pressure ventilation) at birth – Some babies need cardiac compressions and/or IV Adrenaline – Aim for normothermia until the baby meets the inclusion criteria for therapeutic hypothermia – Monitor temperature to avoid hyperthermia
**Anticonvulsants**	– Experts recommend treating neonatal seizures, which are common in HIE and suspected to be an independent cause of brain injury – Obtaining serum levels of antiepileptics, particularly in the first 72 hours if redosing is needed, should be strongly considered.	– Not Mentioned.
**Sedation**	– A low infusion of morphine (≤10 mcg/kg/h) or equivalent opioid is recommended as the initial approach for easing discomfort.	– If the baby shows any signs of distress or there is excessive shivering causing difficulties maintaining the desired baby temperature, consider: ○ Low dose morphine and/or midazolam ○ Paracetamol
**Allopurinol, Xenon, Melatonin, Erythropoietin, Neural Stem Cells and Magnesium Sulphate**	– There is insufficient evidence to recommend their use at this time	– Not Mentioned.
**Therapeutic Hypothermia & Target Temp.**	– Selective head cooling can be achieved with cooling caps fitted around an infant’s head, with the aim of maintaining fontanelle temperature below 30°C. A rectal temperature of 34°C ± 0.5°C – Whole body cooling to a rectal temperature of 33.5°C ± 0.5°C – Whole body cooling is recommended preferentially – The optimal rectal or esophageal temperature appears to be 33.5°C ± 0.5°C for whole body cooling and 34.5°C ± 0.5°C for selective head cooling	– Target a temperature of between 33.0 °C and 34.0 °C
**Duration**	– 72 hours	– 72 hours
**Rewarm**	– Rewarm over 6 to 12 hours (0.5°C every 1 to 2 hours)	– Rewarm baby at a rate not exceeding 0.5 °C every 2 hours
**Brain MRI timing**	– In the absence of an MRI-compatible isolette and other specialized equipment, it is recommended to obtain an MRI once rewarming has taken place, on day of life 4 or 5 – Consider a repeat MRI between days 10 and 14 of life when the imaging and clinical features are discordant or when diagnostic ambiguity persists	– Routinely perform at 7 (5–10) days of life – Cranial US: Perform on day 1 to exclude neurosurgical cause for HIE or structural brain abnormality
**Follow up**	– Follow-up of infants who received hypothermia to a minimum of 2 years, but ideally until school age, in a neonatal follow-up clinic, is recommended.	– Babies with moderate to severe HIE or who received TH require a neurodevelopmental review by early intervention specialists. – Enroll babies with moderate to severe HIE into a standardized follow-up program from birth to 2 years of age. – Babies with mild HIE also require neurodevelopmental follow up.
**Guideline Implementation Tools**	– Not Mentioned.	*Flowcharts and posters:* – Clinical features, investigations, and management – Criteria for therapeutic hypothermia (Cooling) – Checklist for therapeutic hypothermia (cooling) – Passive cooling *Education* – Slide Presentation – Knowledge assessment *Appendices* – Points for discussion with parents – Assessment of encephalopathy severity (Sarnat scoring) – Sarnat and Sarnat staging of HIE *Consumer information*

## Discussion

The difficulty of variation in their quality and evidence base continues despite the significant volume of national and international neonatal CPGs that are regularly released. To our knowledge, this evaluation is unique in that it uses AGREE II to comprehensively assess the quality of recently published HIE CPGs as a part of a nationwide CPG adaptation program (11).

The methodological rigor of the two HIE CPGs was evaluated using the AGREE II instrument, which identified a number of opportunities for improvement. Although the evaluation of the overall guideline quality and the usage of the recommendation are essential sections of AGREE II, it's likely that they are not clearly communicated in the published CPGs' methodology.

A recently published network meta-analysis compared the effectiveness and safety of different neuroprotective interventions for neonates with HIE (23). This study supports the current CPGs that recommend hypothermia for neonates with HIE, regardless of setting. Its findings support whole-body hypothermia as a first-line treatment option due to its ease of use, improved mortality, and positive neurodevelopmental outcomes (23).

Shipley et al. emphasized the importance of training, equipping, and supporting neonatal centers that lack immediate access to cooling services with active therapeutic hypothermia should be prioritized, reducing interruptions in initiating and achieving appropriate target temperature before transfer to specialized tertiary cooling neonatal centers (24).

We continue to recommend compiling the findings of this study with similar quality appraisals of neonatology CPGs to set up a CPGs hub or Recommendation map that would be of the utmost value for healthcare providers caring for newborn babies in selecting and implementing high-quality evidence-based CPGs and recommending them to their colleagues (11, 25–29).

Moreover, we are looking forward to having new evidence-based recommendations in the next editions of these CPGs based on the mounting evidence addressing the new options of care like the use of conventional electroencephalography (EEG) or Amplitude-integrated Electroencephalogram (aEEG) monitoring in neonatal HIE (30, 31).

### Implications for neonatal practice

The findings of our review can be further used to guide all relevant CPG development or adaptation project for neonatal HIE.

Implementation of the results of this systematic review through identifying and selecting high-quality CPGs will decrease variation in the management of neonatal HIE babies which might lead to improved clinical outcomes and decrease legal litigations.

### Implications for future CPG research

We recommend monitoring the implementation of the process and auditing the process for quality improvement. Reporting adverse outcomes or variance of practice might help for future updates.

## Conclusions

The methodological quality of the QMN CPG was superior followed by CPS. Recommendations addressed inclusion and exclusion criteria, fluid management, feeding, resuscitation, anticonvulsant drugs, sedation, allopurinol, xenon, melatonin, erythropoietin, neural stem cells, erythropoietin, magnesium sulphate, therapeutic hypothermia and target temperature, rewarming, brain MRI timing, and follow up.

## Data Availability

The original contributions presented in the study are included in the article/Supplementary Material, further inquiries can be directed to the corresponding author/s.
