# Supplementary material for: Clinical practice guidelines for neonatal hypoxic-ischemic encephalopathy: A systematic review using the appraisal of guidelines for research and evaluation (AGREE) II instrument
Source: Front Pediatr. 2023 Mar 22;11:1092578. doi: 10.3389/fped.2023.1092578 (PMC10073446; doi:10.3389/fped.2023.1092578)
Supplement: Supplementary file 1 [file Datasheet1.pdf]

Supplementary Material

1 Supplementary Data

1.1 Supplement 1. Search strategy

Keywords: neonat*, newborn, infant*, full term, later preterm, hypoxic ischemic encephalopathy, neonatal hypoxia, neonatal asphyxia, birth asphyxia, therapeutic hypothermia, neonatal encephalopathy, encephalopathy, moderate hypothermia, cooling, cooling therapy, total body cooling, head cooling, clinical practice guidelines, guideline*, statement.

List of the websites and databases we searched

CPG databases and libraries:

1. Guidelines International Network (GIN) International Guidelines Library.
    - https://g-i-n.net/international-guidelines-library/
2. ECRI Guidelines Trust (USA). https://guidelines.ecri.org/
3. National Institute of Clinical and Health Excellence (NICE) UK. http://www.nice.org.uk/guidance/
4. Scottish Intercollegiate Guidelines Network (SIGN) UK. http://www.sign.ac.uk/guidelines/
5. EBSCO DynaMed (USA) https://www.dynamed.com/ (subscription required)

Bibliographic databases:

1. PubMed/ MEDLINE https://pubmed.ncbi.nlm.nih.gov/
2. Embase https://www.embase.com/landing?status=grey (subscription required)
3. CINAHL https://www.ebsco.com/products/research-databases/cinahl-complete

Specialized professional societies:

1. American Academy of Pediatrics (AAP) https://www.aap.org/
2. Canadian Paediatric Society (CPS) https://www.cps.ca/
3. British Association of Perinatal Medicine (BAPM) https://www.bapm.org/
4. Royal College of Paediatrics and Child Health (RCPCH) https://www.rcpch.ac.uk/
5. Saudi Neonatology Society (SNS) https://sns.med.sa/

1.1.1 Search strategy (MEDLINE)

Database: Ovid MEDLINE(R) and Epub Ahead of Print, In-Process, In-Data-Review & Other Non-Indexed Citations and Daily <1946 to March 12, 2021>
Search Strategy:
----

1 exp clinical pathway/ or exp clinical protocol/ or exp consensus/ or exp consensus development conference/ or exp consensus development conferences as topic/ or critical pathways/ or exp guideline/ or guidelines as topic/ or exp practice guideline/ or practice guidelines as topic/ or health planning guidelines/ or (guideline or practice guideline or consensus development conference or consensus development conference, NIH).pt. or (position statement* or policy statement* or practice parameter* or best practice*).ti,ab,kf,kw. or (standards or guideline or guidelines).ti,kf,kw. or ((practice or treatment* or clinical) adj guideline*).ab. or (CPG or CPGs).ti. or consensus*.ti,kf,kw. or consensus*.ab. /freq=2 or ((critical or clinical or practice) adj2 (path or paths or pathway or pathways or protocol*)).ti,ab,kf,kw. or recommendat*.ti,kf,kw. or (care adj2 (standard or path or paths or pathway or pathways or map or maps or plan or plans)).ti,ab,kf,kw. or (algorithm* adj2 (screening or examination or test or tested or testing or assessment* or diagnosis or diagnoses or diagnosed or diagnosing)).ti,ab,kf,kw. or (algorithm* adj2 (pharmacotherap* or chemotherap* or chemotreatment* or therap* or treatment* or intervention*)).ti,ab,kf,kw. (648294)
2 exp Infant/ (1159714)
3 (baby or babies or nicu or neaonat* or perinatal or preemi* or permaturity or prematurity or premi or premie? or preterm or pre-term or (low adj birth adj weight) or infan* or newborn? or new-born? or (full adj term) or LBW or VLBW or (("35" or "36" or "37" or "38" or "39" or "40" or "41" or "42" or "43" or "44" or "45") adj5 week?)).mp. (1594848)
4 2 or 3 (1594848)
5 exp Hypoxia/ (80880)
6 (hypoxi? adj1 encephalopath*).mp. (373)
7 (hypoxi? adj1 ((brain adj damag*) or driv*)).mp. (916)
8 ((Brain or anoxic or Cerebr*) adj3 (Hypoxi? adj Ischemi*)).mp. (7314)
9 (hypoxia adj2 (warning or diffus*)).mp. (146)
10 ((Oxygen adj1 Deficien*) or Hypoxemia? or Anoxia? or anoxemia? or HIE or NE).mp. (73287)
11 Brain Ischemia/ (54193)
12 (Ischemi* adj1 (brain or cerebral or Encephalopath*)).mp. (82432)
13 Asphyxia Neonatorum/ (7761)
14 ((Asphyxia adj2 (Neonat* or birth)) or (Respirat* adj3 failure)).mp. (45260)
15 Cryotherapy/ (5235)
16 (Cryotherap* or Cryo-therap* or ((cold or cool* or freez*) adj3 (bath or therap*))).mp. (11902)
17 Cryosurgery/ (13219)
18 (Cryosurger* or Cryoablation? or Cryo-surger* or Cryo-ablation? or (cryoballoon adj abalat*) or (cryogenic adj surger*)).mp. (15579)
19 Hypothermia, Induced/ (20786)
20 (((Induced or therapeutic or moderate or mild or artificial or extracorporeal) adj3 Hypothermia?) or (Targeted adj Temperature adj Manag*) or (refrigeration adj an?esthesia) or (artificial adj hibernat*)).mp. (27832)

21 Cerebrovascular Disorders/ (46824)
22 (Cerebrovascular adj1 (Disease? or Disorder?)).mp. (64508)
23 or/5-22 (360255)
24 1 and 4 and 23 (1058)
25 exp Erythropoietin/ (23760)
26 exp Phenobarbital/ (18616)
27 Morphine/ (38528)
28 (anpec or duromorph or epimorph or miro or morfin or morfine or morphia or morphin or morphine or morphinium or morphium or opso or skenan or adonal or aephenal or agrypnal or alepsal or amylofene or andral or aparoxal or aphenylbarbit or aphenyletten or atrofen or austrominal or barbapil or barbellen or barbenyl or barbilettae or barbilixir or barbinal or barbiphen or barbiphenyl or barbivis or barbonal or barbonalett or barbophen or bardorm or bartol or bialminal or calmetten or calminal or carbronal or cardenal or cemalonal or codibarbital or coronaletta or cratecil or damoral or dezibarbitur or dormina or dormiral or dromural or ensobarb or ensodorm or epanal or epidorm or epilol or episedal or epsylone or eskabarb or etilfen or euneryl or fenbital or fenemal or fenobarbital or fenolbarbital or fenosed or fenylettae or gardenal or gardepanyl or glysoletten or haplopan or haplos or helional or hennoletten or hypnaletten or hypnolone or hypnotal or hypnotalon or hysteps or lefebar or leonal or lephebar or lepinal or lethyl or linasen or liquital or lixophen or lubergal or lubrokal or lumesettes or lumesyn or luminale or luminaletas or luminalette or luminaletten or luminalettes or luminalum or lumofridetten or luphenil or luramin or menobarb or molinal or neurobarb or nirvonal or noptil or nunol or parkotal or pharmetten or phenobal or phenobarb or phenobarbital or phenaemal or phenemal or phenobarbitol or phenobarbiton or phenobarbitone or phenobarbitural or phenobarbyl or phenonyl or phenotal or phenoturic or phenoyl or phenylethylmalonylurea or phenyletten or phenyral or polcominal or promptonal or sedabar or sedicat or sedizorin or sedlyn or sedofen or sedonal or sedonettes or seneval or sevenal or somnolens or somnoletten or somnosan or somonal or spasepilin or starifen or starilettae or stental or teolaxin or theolaxin or triabarb or tridezibarbitur or uni-feno or versomnal or wakobital or zadoletten or zadonalOR erthropoietin or erythropoietin or hematopoietin or hemopoietin).mp. (118241)
29 ("3, 6 dihydroxy n methyl 4, 5 epoxy 7 morphinene" or "microcrystalline morphine suspension" or "trans morphine" or "5 ethyl 5 phenylbarbituric acid").mp. (10)
30 ("hypno tablinetten" or "hypno-tablinetten" or "hypnogen fragner" or "luminal (drug)" or "luminal sodium" or "nova pheno" or "nova-pheno" or "phen bar" or "phenethylbarbital sodium" or "phenobarbital sodium" or "phenyl ethyl barbituric acid" or "phenylethyl barbituric acid" or "phenylethylbarbituric acid" or "phenylethylmalonyl urea" or "seda tablinen" or "sodium phenobarbital" or "sodium phenobarbitone" or "sombutol mcclung").mp. (623)
31 ("11096-26-7" or Erythropoietin or "113427-24-0" or "64fs3bfh5w" or (Alfa adj2 epoetin) or binocrit or hx575 or epogen or eprex or heberitro or Procrit or "SDZ 202-250" or "SDZ 202 250" or SDZ 202250 or SDZ202-250 or "SDZ202 250" or SDZ202250 or "MS Contin" or "Oramorph SR" or Duramorph or Liskantin or Mylepsinum or Resimatil or Desoxyphenobarbital or Mizodin or Mysoline or Apo-Primidone or Apo Primidone or Primaclone or Sertan or Misodine or Primidon).mp. (32464)
32 PERSISTENT FETAL CIRCULATION SYNDROME/ (1187)
33 ("erythropoiesis stimulating factor" or "erythropoietic factor" or "erythropoietic stimulation factor" or "persistent fetal circulation" or "persistent fetus circulation" or "persistent foetal circulation" or "persistent pulmonary hypertension" or PPHN).mp. (2737)
34 or/25-33 (121833)
35 1 and 4 and (23 or 34) (1322)

****

1.1.2 Search strategy (EMBASE)

No.,Query,Results,Date

#44,"#16 AND #22 AND (#41 OR #43)",1998,21 Mar 2021

#43,"'morphine'/de OR '3, 6 dihydroxy n methyl 4, 5 epoxy 7 morphinene' OR 'anpec' OR 'cis morphine' OR 'duromorph' OR 'epimorph' OR 'microcrystalline morphine suspension' OR 'miro' OR 'morfin' OR 'morfine' OR 'morphia' OR 'morphin' OR 'morphine' OR 'morphine alkaloid' OR 'morphine chlorhydrate' OR 'morphine chloride' OR 'morphine hydrochloride' OR 'morphine suspension' OR 'morphinium' OR 'morphium' OR 'opso' OR 'skenan' OR 'trans morphine' OR 'phenobarbital'/de OR '5 ethyl 5 phenylbarbituric acid' OR 'adonal' OR 'aephenal' OR 'agrypnal' OR 'alepsal' OR 'amylofene' OR 'andral' OR 'aparoxal' OR 'aphenylbarbit' OR 'aphenyletten' OR 'atrofen' OR 'austrominal' OR 'barbapil' OR 'barbellen' OR 'barbenyl' OR 'barbilettae' OR 'barbilixir' OR 'barbinal' OR 'barbiphen' OR 'barbiphenyl' OR 'barbivis' OR 'barbonal' OR 'barbonalett' OR 'barbophen' OR 'bardorm' OR 'bartol' OR 'bialminal' OR 'calmetten' OR 'calminal' OR 'carbronal' OR 'cardenal' OR 'cemalonal' OR 'codibarbital' OR 'coronaletta' OR 'cratecil' OR 'damoral' OR 'dezibarbitur' OR 'dormina' OR 'dormiral' OR 'dromural' OR 'ensobarb' OR 'ensodorm' OR 'epanal' OR 'epanal 2' OR 'epidorm' OR 'epilol' OR 'episedal' OR 'epsylone' OR 'eskabarb' OR 'etilfen' OR 'euneryl' OR 'fenbital' OR 'fenemal' OR 'fenemal nm pharma' OR 'fenobarbital' OR 'fenolbarbital' OR 'fenosed' OR 'fenylettae' OR 'gardenal' OR 'gardenal sodium' OR 'gardenale' OR 'gardepanyl' OR 'glysoletten' OR 'haplopan' OR 'haplos' OR 'helional' OR 'hennoletten' OR 'hypnaletten' OR 'hypno tablinetten' OR 'hypno-tablinetten' OR 'hypnogen fragner' OR 'hypnolone' OR 'hypnotal' OR 'hypnotalon' OR 'hysteps' OR 'lefebar' OR 'leonal' OR 'leonal leo' OR 'lephebar' OR 'lepinal' OR 'lethyl' OR 'linasen' OR 'liquital' OR 'lixophen' OR 'lubergal' OR 'lubrokal' OR 'lumesettes' OR 'lumesyn' OR 'luminal (drug)' OR 'luminal sodium' OR 'luminale' OR 'luminaletas' OR 'luminalette' OR 'luminaletten' OR 'luminalettes' OR 'luminalum' OR 'lumofridetten' OR 'luphenil' OR 'luramin' OR 'menobarb' OR 'molinal' OR 'neurobarb' OR 'nirvonal' OR 'noptil' OR 'nova pheno' OR 'nova-pheno' OR 'nunol' OR 'parkotal' OR 'pharmetten' OR 'phen bar' OR 'phenaemal' OR 'phenemal' OR 'phenethylbarbital sodium' OR 'phenobal' OR 'phenobarb' OR 'phenobarbital' OR 'phenobarbital 2' OR 'phenobarbital i' OR 'phenobarbital sodium' OR 'phenobarbitol' OR 'phenobarbiton' OR 'phenobarbitone' OR 'phenobarbitone sodium' OR 'phenobarbitural' OR 'phenobarbyl' OR 'phenonyl' OR 'phenotal' OR 'phenoturic' OR 'phenoyl' OR 'phenyl ethyl barbituric acid' OR 'phenylethyl barbituric acid' OR 'phenylethylbarbituric acid' OR 'phenylethylmalonyl urea' OR 'phenylethylmalonylurea' OR 'phenyletten' OR 'phenyral' OR 'polcominal' OR 'promptonal' OR 'seda tablinen' OR 'sedabar' OR 'sedicat' OR 'sedizorin' OR 'sedlyn' OR 'sedofen' OR 'sedonal' OR 'sedonettes' OR 'seneval' OR 'sevenal' OR 'sodium phenobarbital' OR 'sodium phenobarbitone' OR 'sombutol mcclung' OR 'somnolens' OR 'somnoletten' OR 'somnosan' OR 'somonal' OR 'spasepilin' OR 'starifen' OR 'starilettae' OR 'stental' OR 'teolaxin' OR 'theolaxin' OR 'triabarb' OR 'tridezibarbitur' OR 'uni-feno' OR 'versomnal' OR 'wakobital' OR 'zadoletten' OR 'zadonal' OR 'erythropoietin'/de OR 'erthropoietin' OR 'erythropoiesis stimulating factor' OR 'erythropoietic factor' OR 'erythropoietic stimulation factor' OR 'erythropoietin' OR 'hematopoietin' OR 'hemopoietin' OR 'kidney erythropoietic factor' OR 'renal erythropoietic factor' OR 'persistent pulmonary hypertension'/de OR 'persistent fetal circulation' OR 'persistent fetal circulation syndrome' OR 'persistent fetus circulation' OR 'persistent foetal circulation' OR 'persistent foetal circulation syndrome' OR 'persistent pulmonary hypertension'",267779,21 Mar 2021

#42,"#16 AND #22 AND #41",1393,21 Mar 2021

#41,"#23 OR #24 OR #25 OR #26 OR #27 OR #28 OR #29 OR #30 OR #31 OR #32 OR #33 OR #34 OR #35 OR #36 OR #37 OR #38 OR #39 OR #40",604485,21 Mar 2021

#40,"'cerebrovascular disease'/de OR ((cerebrovascular NEAR/1 (disease$ OR disorder$)):ti,ab,kw)",83395,21 Mar 2021

#39,"'induced hypothermia'/de OR (((induced OR therapeutic OR moderate OR mild OR artificial OR extracorporeal) NEAR/3 hypothermia$):ti,ab,kw) OR 'targeted temperature manag*':ti,ab,kw OR 'refrigeration an$esthesia':ti,ab,kw OR 'artificial hibernat*':ti,ab,kw",25879,21 Mar 2021

#38,"(cryogenic NEXT/1 surger*):ti,ab,kw",88,21 Mar 2021

#37,"(cryoballoon NEXT/1 abalat*):ti,ab,kw",0,21 Mar 2021
#36,"cryosurger*:ti,ab,kw OR cryoablation?:ti,ab,kw OR 'cryo-surger*':ti,ab,kw OR 'cryo-ablation':ti,ab,kw",5313,21 Mar 2021
#35,"cryotherap*:ti,ab,kw OR 'cryo therap*':ti,ab,kw OR (((cold OR cool* OR freez*) NEAR/3 (bath OR therap*)):ti,ab,kw)",13524,21 Mar 2021
#34,"'cryotherapy'/de OR 'cryoablation'/de OR 'cryosurgery'/de",36661,21 Mar 2021
#33,"((asphyxia NEAR/2 (neonat* OR birth)):ti,ab,kw) OR ((respirat* NEAR/3 failure):ti,ab,kw)",65883,21 Mar 2021
#32,"(ischemi* NEAR/1 (brain OR cerebral OR encephalopath*)):ti,ab,kw",62118,21 Mar 2021
#31,"((oxygen NEAR/1 deficien*):ti,ab,kw) OR hypoxemia:ti,ab,kw OR anoxia:ti,ab,kw OR anoxemia:ti,ab,kw OR hie:ti,ab,kw OR ne:ti,ab,kw",92819,21 Mar 2021
#30,"(hypoxia NEAR/2 (warning OR diffus*)):ti,ab,kw",207,21 Mar 2021
#29,"((brain OR anoxic OR cerebr*) NEAR/3 'hypoxic ischemic'):ti,ab,kw",2868,21 Mar 2021
#28,"((brain OR anoxic OR cerebr*) NEAR/3 'hypoxia ischemic'):ti,ab,kw",48,21 Mar 2021
#27,"((brain OR anoxic OR cerebr*) NEAR/3 'hypoxia ischemia'):ti,ab,kw",1297,21 Mar 2021
#26,"((brain OR anoxic OR cerebr*) NEAR/3 'hypoxic ischemia'):ti,ab,kw",96,21 Mar 2021
#25,"(hypoxi? NEAR/1 ('brain damag*' OR driv*)):ti,ab,kw",1388,21 Mar 2021
#24,"(hypoxi? NEAR/1 encephalopath*):ti,ab,kw",618,21 Mar 2021
#23,"'hypoxia'/de OR 'brain hypoxia'/de OR 'newborn hypoxia'/de OR 'hypoxemia'/de OR 'anoxia'/de OR 'brain ischemia'/de OR 'hypoxic ischemic encephalopathy'/de",334535,21 Mar 2021
#22,"#18 OR #19 OR #20 OR #21",1004808,21 Mar 2021
#21,"'full term':ti,ab,kw OR 'lbw':ti,ab,kw OR 'vlbw':ti,ab,kw",33332,21 Mar 2021
#20,"infan*:ti,ab,kw OR newborn$:ti,ab,kw OR 'new born$':ti,ab,kw",721711,21 Mar 2021
#19,"baby:ti,ab,kw OR babies:ti,ab,kw OR nicu:ti,ab,kw OR neaonat*:ti,ab,kw OR perinatal:ti,ab,kw OR preemi*:ti,ab,kw OR permaturity:ti,ab,kw OR prematurity:ti,ab,kw OR premi:ti,ab,kw OR premie$:ti,ab,kw OR preterm:ti,ab,kw OR 'pre term':ti,ab,kw OR newborn*:ti,ab,kw OR 'low birth weight':ti,ab,kw",508615,21 Mar 2021
#18,"(('35' OR '36' OR '37' OR '38' OR '39' OR '40' OR '41' OR '42' OR '43' OR '44' OR '45') NEAR/5 week$):ti,ab,kw",130163,21 Mar 2021
#17,"'infant'/exp",1171191,21 Mar 2021
#16,"#1 OR #2 OR #3 OR #4 OR #5 OR #6 OR #7 OR #8 OR #9 OR #10 OR #11 OR #12 OR #13 OR #14 OR #15",1023073,21 Mar 2021
#15,"(algorithm* NEAR/1 (pharmacotherap* OR chemotherap* OR chemotreatment* OR therap* OR treatment* OR intervention*)):ti,ab,kw",13763,21 Mar 2021
#14,"(algorithm* NEAR/1 (screening OR examination OR test OR tested OR testing OR assessment* OR diagnosis OR diagnoses OR diagnosed OR diagnosing)):ti,ab,kw",5419,21 Mar 2021
#13,"(care NEAR/1 (standard OR path OR paths OR pathway$ OR map$ OR plan$)):ti,ab,kw",39628,21 Mar 2021
#12,"recommendat*:ti,kw",56724,21 Mar 2021
#11,"((critical OR clinical OR practice) NEAR/1 (path OR paths OR pathway OR pathways OR protocol*)):ti,ab,kw",20341,21 Mar 2021

#10,"consensus*:ti,ab,kw",235694,21 Mar 2021
#9,"cpg:ti OR cpgs:ti",7095,21 Mar 2021
#8,"((practice OR treatment* OR clinical) NEXT/1 guideline*):ab",65297,21 Mar 2021
#7,"standards:ti,kw OR guideline:ti,kw OR guidelines:ti,kw",161665,21 Mar 2021
#6,"'position statement*':ti,ab,kw OR 'policy statement*':ti,ab,kw OR 'practice parameter*':ti,ab,kw OR 'best practice*':ti,ab,kw",52717,21 Mar 2021
#5,"guideline:it OR consensus:it",0,21 Mar 2021
#4,"'practice guideline'/exp",588166,21 Mar 2021
#3,"guideline:it OR 'practice guideline':it OR 'consensus development conference':it OR 'consensus development conference, nih':it",0,21 Mar 2021
#2,"'clinical pathway'/exp OR 'clinical protocol'/exp OR 'consensus'/exp",188896,21 Mar 2021
#1,"'guideline'/exp",141,21 Mar 2021

Number of retrieved results

Medline 1,322, Embase 2,400, CINAHL 783 (Total 4,505 results) – EndNote removed 2,016 duplicates ---

Total: 2489

Results of CPG databases search

- **CPG Databases:** GIN Library (n =0), ECRI Guidelines Trust (n=1), DynaMed (n=11), NICE (n=1), and SIGN (n=0)
- **Professional Societies:** AAP (n =2), CPS (n=1), RCPC (n=1), BAPM (n=1), SNS (n=0). Citation searching (n =0)

2 Supplementary Tables

2.1 Supplement Table for interpretation of the strength of agreement according to K value.

Value of K	Strength of agreement
< 0.20	Poor
0.21 - 0.30	Fair
0.31 - 0.40	Moderate
0.41 - 0.60	Good
0.61 - 0.80	Very good
0.81 – 1.00	Excellent

2.2 Supplement Table for Classification of the strength of agreement among the four raters against the two HIE clinical practice guidelines

	Poor	Fair	Good	Very good	Excellent	Sum of scores	Sum of OA 1 scores	Overall assessment (OA 1)
**1. CPS 2018 Position Statement**	0	0	12	6	6	333	19	Very Good
**2. QMNCG 2021 Queensland Maternity and Neonatal Clinical Guideline**	0	0	2	6	16	431	24	Excellent
